# Lack of experience-based stratification in homing pigeon leadership hierarchies

**DOI:** 10.1098/rsos.150518

**Published:** 2016-01-13

**Authors:** Isobel Watts, Benjamin Pettit, Máté Nagy, Theresa Burt de Perera, Dora Biro

**Affiliations:** 1 Department of Zoology, University of Oxford, Oxford OX1 3PS, UK; 2 MTA-ELTE Statistical and Biological Physics Research Group, Hungarian Academy of Sciences, Budapest, Hungary; 3 Department of Biological Physics, Eötvös University, Budapest, Hungary

**Keywords:** *Columba livia*, hierarchy, leadership, navigational experience, pigeon, collective motion

## Abstract

In societies that make collective decisions through leadership, a fundamental question concerns the individual attributes that allow certain group members to assume leadership roles over others. Homing pigeons form transitive leadership hierarchies during flock flights, where flock members are ranked according to the average time differences with which they lead or follow others' movement. Here, we test systematically whether leadership ranks in navigational hierarchies are correlated with prior experience of a homing task. We constructed experimental flocks of pigeons with mixed navigational experience: half of the birds within each flock had been familiarized with a specific release site through multiple previous releases, while the other half had never been released from the same site. We measured the birds' hierarchical leadership ranks, then switched the same birds' roles at a second site to test whether the relative hierarchical positions of the birds in the two subsets would reverse in response to the reversal in levels of experience. We found that while across all releases the top hierarchical positions were occupied by experienced birds significantly more often than by inexperienced ones, the remaining experienced birds were not consistently clustered in the top half—in other words, the network did not become stratified. We discuss our results in light of the adaptive value of structuring leadership hierarchies according to ‘merit’ (here, navigational experience).

## 1. Introduction

During collective travel, individuals must maintain cohesion in order to sustain the benefits associated with group motion, including predator avoidance, increased foraging benefits and enhanced navigational efficiency [1]. Groups are rarely homogeneous; inter-individual differences in knowledge, motivation or social status may be present, resulting in potential conflicts of interest. When conflicts arise, leader/follower interactions can emerge from these inter-individual differences [2]. A fundamental question of group living is how individual attributes allow certain group members to assume leadership roles over others. In systems where leadership is implicated, the benefits derived from, and ultimately the adaptive value of, collective decision-making will depend on the mechanism that places specific individuals into leadership positions.

Theoretical and empirical work suggests a variety of inter-individual differences can affect specific individuals' abilities to influence group movements [3,4]. For example, dominance has been shown to be associated with leadership in many mammalian species with strong social ties, including grey wolves (*Canis lupus*), chacma baboons (*Papio ursinus*), rhesus macaques (*Macaca mulatta*) and beef cows (*Bos taurus*) [5–8]. Furthermore, observations suggest that leadership is assumed by knowledgeable individuals in golden shiners (*Notemigonus crysoleucas*) and pairs of homing pigeons (*Columba livia*) [4,9], or by the oldest individuals in elephants (*Loxodonta africana*) and dogs (*Canis canis*) [10,11] (with the caveat that age is typically not independent of knowledge and experience). Empirical evidence also suggests a role for motivation, resulting in leadership ‘according to need’ [3]. For example, frontal positions during group movements are taken up by hungrier individuals in several fish species [12,13] and by lactating females in plains zebras (*Equus burchellii*) [14]. In groups where knowledge and motivation are similar across individuals, intrinsic factors such as temperament (boldness/shyness) have been shown to predict leadership in a range of species [13,15–17]. Overall, however, it is important to note that these factors are not mutually exclusive, nor are they likely to operate independently. Instead, the mechanisms behind the emergence of leader/follower interactions are most likely to involve a combination of factors specific to each system. For example, when pairs of three-spined sticklebacks (*Gasterosteus aculeatus*) have similar nutritional levels, bolder individuals initiate leading events. However, when nutritional levels vary, the individual with the lower nutritional state initiates leaving cover [13].

Homing pigeons are an excellent model species for the study of collective decision-making due to their gregarious nature and the ease with which inter-individual differences within flocks can be manipulated. Early studies based on direct observation suggested that leadership, by one or a few birds, was operating in homing pigeon flocks ([18]; see also [19]). With recent technological advances, birds' movements within the flock can now be measured through on-board GPS loggers, yielding high-resolution data on collective movement dynamics and allowing more detailed insights into leadership in these flocks. Such studies have revealed that pigeon flocks form transitive, multi-level navigational leadership hierarchies, with individuals contributing (on average) with different weights to the movement decisions of the flock [20]. The phenomenon itself appears robust, observable in flocks of different sizes [21] and in different populations of pigeons [20–22]. A recent study [23] has confirmed individual flight speed as an important factor in structuring the leadership hierarchies, showing faster pigeons tend to lead flocks. Speed, in turn, has been shown to be influenced by morphological factors [23] and motivation [24]. However, the extent to which previous navigational experiences modulate these relationships remains unknown, since all birds in Pettit *et al*.'s study [23] were equal in their lack of prior homing experience. Here, therefore, we focus on the link between leadership and navigational experience—an attribute that (i) is particularly relevant in determining how group performance varies as a function of the identity of the leader(s), and (ii) can be most easily manipulated experimentally.

Differences in knowledge can stem not only from differences in navigational experience through previous encounters with the landscape and other environmental cues, but also from individual differences in navigational skill or strategy. Although there is evidence that experienced birds are more likely to lead when flown in a pair [9], when scaled up to larger flocks of around 10 birds, increases in experience do not improve a bird's chance of leading. In a study by Flack *et al*. [25], existing leadership hierarchies were resistant to change when specific individuals' navigational knowledge was selectively increased through additional solo flight training. One possible reason for the differences in these findings was, as the authors suggested, that their latter experiment failed to generate large enough differences in knowledge to re-organize the hierarchies. Prior to the solo training, all birds had already participated in eight flock flights, and therefore most probably had already approached near-maximal levels of navigational efficiency [26], leading to a ceiling effect.

In this study, we aim to resolve this problem and to examine systematically how a specific individual attribute—navigational experience—contributes to structuring leader–follower relations in large-scale orientation tasks performed by homing pigeon flocks. We constructed two flocks of mixed navigational experience, in which half the birds were highly familiar with a specific release site while the other half were locally inexperienced (having never been released from the same site previously), and then repeated the design at a second site where the roles of the two halves in each group were reversed. We tracked flock flights from both sites using miniature GPS devices carried by all subjects, and then compared the hierarchical ranks obtained by the same individuals at the two sites. Our rationale was that if leadership was influenced by navigational experience, then the results should reveal clear differences in the ranks attained by birds at the two sites, depending on whether they belonged to the experienced or the inexperienced half of the flock. Essentially, at each site, we predicted a form of ‘stratification’ within the leadership network based on knowledge, with the more experienced birds consistently clustered together in the top half of the hierarchy, and less experienced birds clustered in the bottom half. Crucially, individual birds' occupancy of the two clusters would be expected to reverse between sites, providing a clear diagnostic of experience-based structuring in the network.

## 2. Material and methods

### 2.1 Subjects

This study used 27 adult homing pigeons bred and housed in two lofts, of around 140 birds, at the University of Oxford Field Station at Wytham (51°6′58.34′′ N, 1°19′02.40′′ W). Throughout the experiment, birds had free access to food (standard homing pigeon grain mix), water, minerals and grit. All experimental birds were 2 years old and all had received basic training consisting of multiple flock and solo releases from sites 2–3 km from the loft. In addition, 23 of the 27 birds had experimental homing experience: the previous year they had flown both in solo and paired flights from a single release site (Kirtlington; distance to home: 10.45 km, direction to home: 196.4°), but none had ever visited the two release sites used in the current experiment.

Twenty of the Kirtlington-experienced birds were randomly selected and divided into four groups of five individuals (henceforth referred to as groups A, B, C and D). The remaining seven birds were assigned to a fifth flock (‘reserves’), which also received training in parallel with flocks A–D, and from which we transferred birds to experimental flocks when any of the experimental birds were lost. This occurred in four cases: one bird in group A, one bird in group C and two birds in group D.

### 2.2 Experimental procedure

Two release sites were used: Site 1 near High Cogges (distance to home loft: 9.36 km, direction to home loft: 90.1°) and Site 2 near Beckley (10.11 km, 264.3°). Based on previous studies, these distances—approximately 10 km in each case—were deemed far enough from the loft that we could assume that birds were initially navigationally inexperienced at our test sites. Several observations are relevant to this assumption. First, numerous past studies observed that birds that had not previously been released from sites as little as 5 km from their loft show initially steep learning curves in that they gradually improve their efficiency and route fidelity over a series of releases before these measures asymptote around the 8th–10th flight (an effect first reported in [27]; see also review in [26]). Releasing birds a further 10–20 times causes either no or only limited increase in the efficiency of routes. Both of these are highly robust effects, reproduced reliably in every experiment so far conducted under comparable conditions to ours [26]. Second, data from feral urban pigeons shows only 7.5% were documented to fly over 2 km during foraging trips [28], suggesting that even birds that do not have an ad libitum supply of food at their home loft or roost (as is the case with our subjects) do not typically undertake foraging or exploratory journeys long enough to bring them even halfway to our release sites. Third, while there is a possible alternative to a purely learning-based account—i.e. that training causes a switch in navigational strategy rather than, or as well as, learning (see Discussion)—in either case, considerable differences are evident in homing performance as a function of flight number. In sum, training birds from previously unvisited sites has clear, observable effects on birds' homing, and these effects can be ascribed to increases in experience.

At Site 1, groups B and C received the ‘Experienced’ treatment (see below for details of experimental treatments), while groups A and D were the ‘Inexperienced’ flocks. The treatments were reversed at Site 2: groups A and D received the ‘Experienced’ treatment and groups B and C the ‘Inexperienced’ treatment.

For the training phase, the Experienced treatment involved the groups being released eight times (T1–T8) in succession at their respective sites, while the Inexperienced groups of the same site were never taken to that site. This ensured that between the two treatment groups, the birds had very different levels of experience. The Site 1 releases and Site 2 releases were carried out in an alternating order, allowing all groups to experience similar weather conditions and time intervals between successive flights.

**Figure 1.**
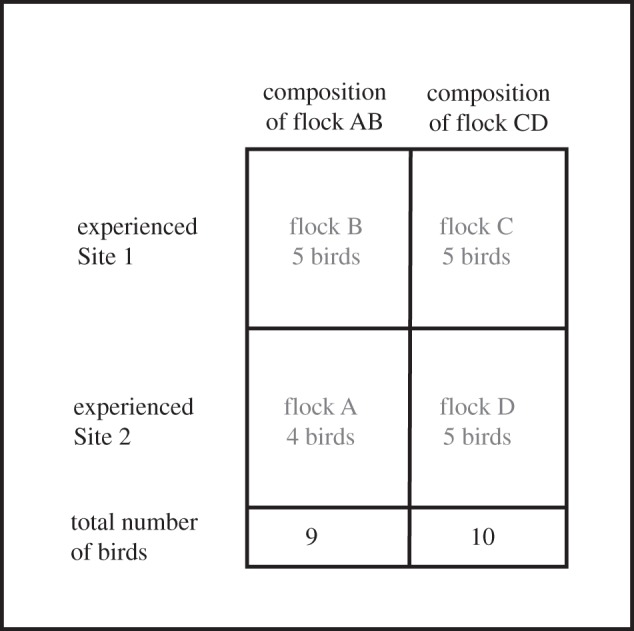
Composition of test flocks (groups AB and CD) with respect to training experienced by their members.

The testing phase followed completion of training. We combined a group of Site 1 Experienced and a group of Site 2 Experienced birds (groups B and A, respectively), and did the same for the remaining two flocks (groups C and D), thereby creating two mixed-experience flocks of 10 (groups AB and CD). The compositions of the two flocks are illustrated in figure 1. Each flock of 10 had eight experimental test releases (E1–E8) from each site, alternating between sites. As one bird from flock A did not return from the first test release, for the remaining test releases group AB consisted of four A-birds and five B-birds.

During both the training and testing phases, releases were carried out on consecutive days when the sun was visible and wind speed was less than 7 m s^−1^. A maximum of two releases were performed each day and there was a minimum interval of 1 h between releases.

### 2.3 Data logging

Subjects were tracked on all training and testing flights using commercially available GPS loggers (Qstarz BT-Q1300ST). These devices weighed 15.5 g and logged time-stamped longitude and latitude coordinates at 5 Hz (absolute spatial error: mean=1.69 m, 95th percentile =4.33 m [29]). They were attached using Velcro strips glued onto clipped feathers along the birds' back. Data were downloaded using QTravel (Qstarz v. 3.2) software and all processing of data was carried out in Matlab (R_2012B) using custom-written code [30]. The geodetic latitude and longitude coordinates provided by the GPS were first converted to X and Y Universal Transverse Mercator (UTM) coordinates using UTM projection. Tracks were speed filtered using the same method as Pettit [30], to remove portions of the tracks when the pigeons had landed, by only keeping points where speed was continuously above 5 m s^−1^ either for 10 s before or 10 s after the time point. Pigeons frequently circle near the start and at the end of a flight, so, to focus on the homing portion of the tracks, only points 100 m from the release point (start) and from the loft (end) were used.

### 2.4 Data analysis

Where possible, we used linear mixed models (LMMs) or generalized linear mixed models (GLMMs) to analyse the data, with the lme4 package in R [31,32]. For all LMMs and GLMMs in this study, we checked the assumptions of linearity, normality and homoskedasticity by visual inspection of plotted residuals.

In order to evaluate the increase in birds' navigational experience through the training phase, we used two measures of homing performance: (i) homing efficiency and (ii) distance of route from the beeline. We defined efficiency as the straight-line distance between the release site and the loft divided by the actual distance travelled by the bird [33] and the distance from the beeline as the nearest-neighbour distance from each point on the track to a straight-line path composed of 100 points from start to finish [29]. These two measurements are often correlated (low efficiency with large distance from the beeline and high efficiency with small distance from the beeline), although not always (a bird may stay close to the beeline but fly a locally tortuous track, leading to low efficiency and small distance from beeline). In order to compare flock homing performance across the training releases, we calculated the groups' mean efficiency and distance from the beeline (excluding birds that split). A bird was deemed to have split if it spent less than 75% of the flight within 100 m of another bird. Using an LMM, with *release* and *site* as fixed factors and *group* as a random factor, we examined how efficiency and distance from the beeline changed as releases progressed from T1 to T8.

To measure leader–follower relationships in each experimental group during testing, we calculated the directional correlation delay for each possible pairing of birds using methods based on Nagy *et al*. [20] and Pettit *et al*. [23]. This analysis measures the temporal relationship between a bird's flight direction and those of the other flock members, identifying birds as followers when they perform changes in direction that match those of others but are delayed in time. The directional correlation between pairs *i* and *j* is *C*_*ij*_(*τ*)=〈*v*_*i*_(*t*)⋅*v*_*j*_(*t*+*τ*)〉, where *v*_*i*_(*t*) is the normalized velocity of *i* at time *t* and *v*_*j*_(*t*+*τ*) is the normalized velocity of bird *j* at time *t*+*τ*. Normalized velocity is calculated by dividing the velocity vector by its magnitude (i.e. *v*_*i*_(*t*)=*x*_*i*_(*t*)′/|*x*_*i*_(*t*)′|). The value of *τ*_*ij*_ (tau) that maximizes the *C*_*ij*_(*τ*) correlation function across *t* is the average time delay between a pair of birds (electronic supplementary material, figure S1). The directional correlation time delay (*τ*_*ij*_) for a pair of birds *i* and *j* is the time taken for bird *i* to react to a change in direction of bird *j*. If *τ*_*ij*_<0, then when bird *i* turns *j* has already turned and therefore can be interpreted as *j* leading *i*. We resolved leader–follower relations between all pairs of birds that spent a minimum of 120 s within 100 m of each other during flight (electronic supplementary material, figure S2).

To calculate unique hierarchical positions for each bird, we calculated the average directional correlation time delay of bird *i* with the rest of the flock (*τ*_*i*_) weighted by duration [23]. The most positive value identifies the bird at the top of the hierarchy. To test individuals' positional consistency in the hierarchy across the testing phase releases (E1–E8), we calculated intra-class correlation coefficients using LMMs, with *individual* and *release* as random effects. This method is identical to that used in Pettit *et al*. [23]. The formula $r=\sigma_{\mathrm{individual}}^{2}/(\sigma_{\mathrm{individual}}^{2}+\sigma_{e}^{2})$, where *σ*^2^_individual_ is the between-group variation and $\sigma_{e}^{2}$ is the residual variation, gives the repeatability measurement as the proportion of variance due to the individual at a particular site [34]. To test for significance, we used a randomization test, where we randomized the 10 (nine in the case of group AB) *τ*_*i*_ values within each release and re-calculated *r* (*r*_rand_) for each iteration. We calculated the *p*-value as the proportion of 10^4^ randomizations with *r*_rand_≥*r* [34].

To test if experience affects hierarchical position in the flock, we calculated for each bird its average position within the hierarchy across the eight test releases ($\tau_{i}^{\mathrm{bar}}$) by averaging the *τ*_*i*_ values across the releases. Using a paired *t*-test, we compared the mean difference in $\tau_{i}^{\mathrm{bar}}$ values obtained when birds flew from the site at which they were experienced and those obtained from the site at which they were inexperienced. As it was on the first release of testing from a given site that the inexperienced birds were maximally naive, we repeated this test for just the first release of testing. In addition, making use of the full dataset of test releases rather than just the per-bird averages as above, we examined whether experienced birds had higher *τ*_*i*_ values in any given release, using an LMM. Finally, looking more specifically at only the birds at the very top and very bottom of the leadership hierarchy, we tested, using binomial tests, whether the highest- or lowest-ranked birds were more likely to belong to one treatment group (experienced or inexperienced, respectively) than to the other.

## 3. Results

### 3.1 Navigational performance

First, we evaluated the effect of training on homing performance. The number of birds used for each release during this analysis is reported in the electronic supplementary material, table S1. Across the eight releases of training (T1–T8), homing efficiency significantly increased by 0.529±0.211 (mean±s.d.), resulting in an average 71% reduction in path length compared to the first release (LMM with *release* as fixed factor and *group* as a random factor: maximum-likelihood comparison to model without release *p*<0.001). Distance from the beeline significantly decreased by an average of 62±33% from the first release (mean±s.d.) (natural logarithm transformation on skewed response variable, LMM with *release* as a fixed factor and *group* as a random factor: maximum-likelihood comparison to model without release *p*=0.034). In both tests, including the fixed factor *site* did not significantly lower the AIC calculated by maximum likelihood and thus did not improve the models' fit so was removed (electronic supplementary material, table S2). These results demonstrate that birds adopted increasingly direct routes home over the course of training, confirming that, while initially inexperienced, training resulted in an increase in their levels of navigational experience.

### 3.2 Leadership

**Figure 2.**
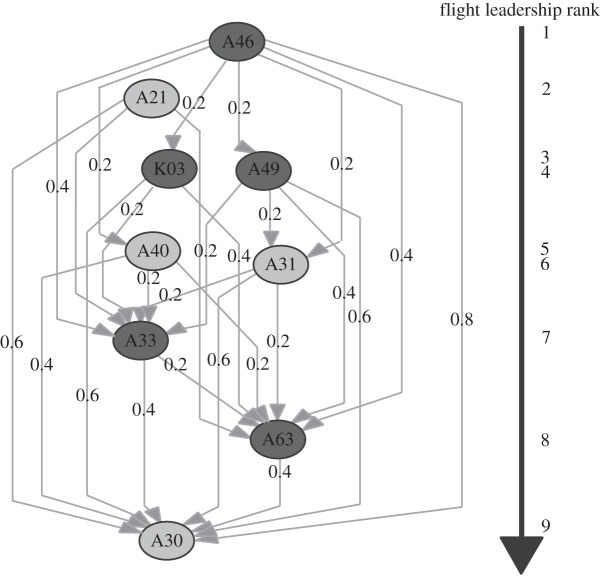
Example of leadership hierarchy. Network shown is for flock AB, site 2, release 7. Nodes are individual birds, and for each pairwise comparison edges point from leader to follower. The values represent the time delays (in seconds) between each pair. The lack of an edge means either the *C*_max_ value between the pair was below 0.99 or the delay was below 0.2, the lowest resolution of the GPS device. Dark grey nodes show individuals experienced at the site shown, while light grey nodes are the inexperienced birds.

Transitive, multi-level leadership hierarchies were detected during our flocks' flights from both sites (see figure 2 for an example). However, for two of the four groups, we found no significant individual repeatability in individuals' average directional correlation delay times across their eight testing releases (table 1), indicating that the leadership hierarchy did not remain consistent across flights. Only at Site 2 was there low but significant repeatability for Groups AB and CD. We did not find evidence of significant repeatability between the two sites for any of the groups (table 1), suggesting that individuals did not maintain consistent ranks when they were tested as experienced versus inexperienced flock members.

**Table 1.** Individual consistency in hierarchical position within leadership networks. *r* is the intra-class correlation coefficient from an LMM with the random effects shown. Six repeatability values were calculated across all eight testing phase releases (E1–E8); four are consistency of leadership among flock flights at a particular site (see top four rows of table) and two are consistency of leadership between sites for both flocks (bottom two rows). *p*-values were calculated using a randomization test as the proportion of 10^4^ randomizations with *r*_rand_≥*r*. Rows in italic represent significant levels of consistency in leadership ranks.

response	random effects	*r*	*p*
hierarchical position ($\tau_{i}^{\mathrm{bar}})$ group AB site 1	individual, release	0.108	0.085
*hierarchical position*$\left(\tau_{i}^{\mathrm{bar}}\right)$*group AB site 2*	*individual*, *release*	*0*.*244*	*0*.*004*
hierarchical position $\left(\tau_{i}^{\mathrm{bar}}\right)$ group CD site 1	individual, release	0.087	0.104
*hierarchical position*$\left(\tau_{i}^{\mathrm{bar}}\right)$*group CD site 2*	*individual, release*	*0*.*234*	*0*.*013*
hierarchical position $\left(\tau_{i}^{\mathrm{bar}}\right)$ group AB	individual, site	0.000	1
hierarchical position $\left(\tau_{i}^{\mathrm{bar}}\right)$ group CD	individual, site	0.420	0.137

Due to the low/non-significant repeatability of individuals' positions in the hierarchy across releases, comparing birds' average time delay values ($\tau_{i}^{\mathrm{bar}}$) across testing (i.e. between sites where they were either experienced or inexperienced) should be evaluated with care. Birds did not have significantly higher mean time delay values at sites where they were experienced, compared with sites where they were inexperienced (Flock 1: paired two-sample *t*-test *t*_9_=0.913, *p*=0.372; Flock 2: paired two-sample *t*-test *t*_10_=−0.923, *p*=0.367). Although these results are based on average measurements across all eight releases, and may thus have been affected by the low repeatability scores across releases, the same results held true even when considering only the first release (Release E1) (Flock 1: paired two-sample *t*-test *t*_9_=1.05, *p*=0.305; Flock 2: paired two-sample *t*-test *t*_10_=−1.49, *p*=0.159). An additional analysis, which controlled for any potential bias due to low repeatability by the LMM taking into account the *τ*_*i*_ values calculated for each individual in every flight rather than averaging across them, further confirmed these results. Experience had no significant effect on the *τ*_*i*_ value of birds within a flock (LMM with *experience* as a fixed factor and *individual* nested in *group* as a random factor: maximum-likelihood comparison to model without *experience* *p*=0.110), showing that there was no stratification in the network based on birds' experience. *site* and *release* were non-significant factors and thus were removed from the model.

**Figure 3.**
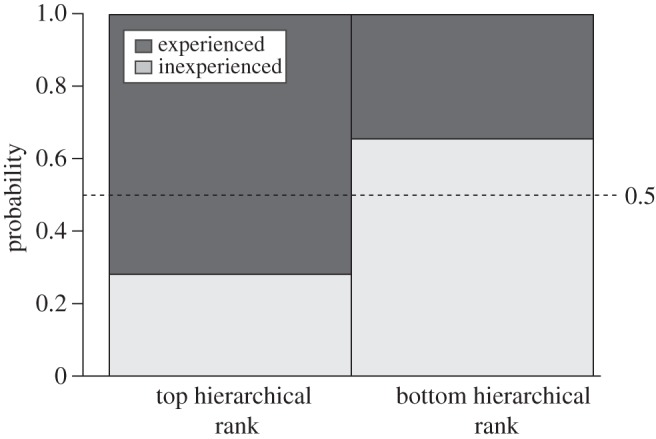
The probability that an individual occupies the top or the bottom leadership hierarchical position as a function of experience. Data are across all flights. Dark grey represents experienced birds and light grey inexperienced birds. The dashed line indicates chance level. Experienced birds occupied the top leadership hierarchical position more often than expected by chance (see main text for statistical detail).

The identity of the bird occupying the highest position in the hierarchy was significantly associated with experience (binomial test, expected =0.5, *p*=0.020). In 23 out of 32 flights (72%), the highest ranked bird was experienced (figure 3). Table 2 shows the number of different experienced birds that led across the 32 flights. In three of the four release series, multiple different birds led, but this was not the case for Group CD at Site 1 where leadership was dominated by just one bird. To examine the effect that this particular bird had on our results, we repeated our analysis after excluding the dataset from Group CD at Site 1. Our conclusions remain largely unchanged (leadership was still assumed by an experienced bird 71% of the time). This trend was strongest for the first two flights, where across all four flocks, the highest ranked bird was always an experienced individual. Furthermore, for three of the four flocks, we did not observe an inexperienced bird occupying the top position before the fifth release. Birds at the bottom of the leadership hierarchy were more often inexperienced than experienced ones (21 inexperienced out of 32 releases; figure 3) but this difference was not significant (binomial test, expected =0.5, *p*=0.055).

**Table 2.** Number of flights led by an experienced bird, and number of different birds that contributed to these counts, in each of the eight-release series performed by the two groups at the two sites. In three of the release series leadership is assumed by multiple different birds; only in one case (group CD at site 1) does one individual dominate all flights. Analyses (see main text) were repeated both with and without the inclusion of the latter dataset.

	group AB site 1	group AB site 2	group CD site 1	group CD site 2
number of flights led by experienced bird	5	7	6	5
number of different experienced birds who led	3	5	1	3

## 4. Discussion

In animal groups in which at least some decisions are made by leaders rather than being fully ‘democratic’ (i.e. rely on unequal rather than equal contributions from all group members), the accuracy of collective decisions will depend on the factors that place specific individuals into leadership positions. For flocks of birds navigating towards a target, for example, the flock can assume a more efficient route if the birds with greater navigational experience contribute with the greatest weight to the flock's movement decisions. Previous research has suggested that experience may indeed be an important individual attribute that affects birds' propensity to lead during flock flights [20], however only in cases where inter-individual differences in experience are sufficiently large [9]. Our present experiments were designed to examine whether the experience effect detected in pairs of birds by the latter study [9] would scale up to larger groups and to the context of leadership hierarchies. We identified an important subtlety: we found that while experience did not consistently affect the majority of positions in the hierarchy (in concordance with [25]), the top rank was predictably assumed by a relatively more experienced bird. This was especially prominent during early releases where the difference in experience between the two subsets was greatest. Overall, our results lend support to the suggestion that experience is an important factor in determining leadership during short-range homing flights, but also imply that additional individual attributes will influence the hierarchical structure of the flock as a whole, typically outweighing—or acting in combination with—experience as we move below the top ranks in the hierarchy.

It is worth noting that while our experimental design relied on large differences in experience between the two treatment groups, with the assumption that the inexperienced group had never previously visited the release sites, we could not be fully certain that none of our birds had spontaneously ranged in the vicinity. As such, we could not guarantee that they were fully naive. However, the steep increases we observed in route efficiency as a function of release number (consistent with numerous previous studies [26]) strongly suggest an increase in birds' familiarity with the local landscape through learning, as a result of training. If we do hypothesize that birds were not fully naive with the sites at the start of training, then it is possible that we were observing—rather than changes in familiarity—changes in navigational strategy (for example, a shift away from sun-compass-based towards more landmark-based navigation [35]). Nonetheless, in either case, the increases we observed in route efficiencies and route recapitulation must be inferred to be the consequence of increases in experience, validating our experimental design.

That experience may be an important factor in structuring leadership hierarchies has a firm theoretical basis. When travelling in groups, not all individuals will possess pertinent navigational information. Theoretical work by Couzin *et al*. [36] has shown that a relatively small proportion of informed individuals are sufficient to guide a group and that information can be transferred without knowledge of which individuals are informed. High navigational certainty may result in some individuals placing greater weight on their own decisions than on those of others or on maintaining group cohesion, resulting in them acting as leaders (whom others follow) and having the greatest input into the groups' movement decisions (see models in [3]). Following an experienced member of the flock can benefit all members as it can increase the group's navigational accuracy, which in turn reduces, among other things, energy expenditure, time spent at risk of predation and time spent away from the nest. We would argue that these benefits are maintained even if only the bird at the top of the leadership hierarchy is experienced, since by definition these birds' knowledge will have the greatest influence on the flock's overall movement.

Given that the effect of navigational experience does not seem to extend beyond the top rank, what other factors may be involved in structuring leadership hierarchies in pigeon flocks? Theoretical and empirical evidence suggests a number of potentially relevant individual attributes. Importantly, the model developed by Pettit *et al*. [29] predicts that, with all else being equal, faster birds—i.e. those that attain higher speeds when flying solo—will fly at the front of flocks and therefore dominate the choice of route. This prediction has now been empirically confirmed in both paired [29] and larger flock flights [23]. However, it is important to note that speed may itself be a function of a variety of factors, including age, morphology, physiology and motivation. In addition, it is currently unknown whether temperament—a parameter that in many species has been shown to influence leadership [13,15–17]—has any impact on the organization of decision-making in pigeon flocks. These factors may act in combination to determine individuals' speed and positioning within the flock, and thus potentially their rank in the hierarchy.

An interesting, and unexpected, feature of our data was that the leadership hierarchy was not stable across releases for two of the four experimental flocks. This is in contrast to previous studies using both GPS-based quantitative analysis [20,23,25] and visual observations [18] which report repeatability in individual birds' relative ranks from flight to flight and even across contexts. Our observed lack of hierarchy stability also gives weight to the suggestion that while individual flight speed (or some correlate thereof) is an important factor in structuring leadership hierarchies [23], it does not act as the sole or primary determinant of an individual's leadership rank, at least under some circumstances. One difference between this and previous studies was the relative homogeneity in the age of the subjects in our flocks: all of our subjects had hatched in the same year, whereas other studies have used mixed-age flocks. While experience naturally positively covaries with age in most animal groups, the role of age in affecting speed in homing pigeons in unknown. Small differences in age have been shown to influence leadership in small flocks but only in very young pigeons (under 1 year) [19]. Santos *et al*. [22] showed that same-aged flocks experienced a reduction in leadership stability (although these authors themselves did not test mixed-age flocks), which could explain the evident lack of stability across releases for two of the four flocks in our study. Another, potentially relevant, difference between our study and previous studies concerns the experimental procedure employed for releases. In previous studies that used multiple sites [20,23,25], all releases were carried out at one site before the experiment moved to another. In contrast, the releases in this study alternated between sites, in order to balance the experience level of the two groups between the two sites. Flack *et al*. [37] showed that gaining experience with multiple routes concurrently (i.e. being released in an alternating fashion from different sites) did not seem to hinder individuals' ability to learn routes. However, the effect of multiple-route learning on the formation and stability of navigational leadership hierarchies is unknown, and needs further investigation. The fact that significant stability was found at one of our sites may suggest that the effect is at least partially site-specific.

In conclusion, our results confirm an important effect of navigational experience on leadership in homing pigeons at the highest, most influential position of the leadership hierarchy. Intuitively, this would appear to make sense from an adaptive perspective: having an experienced bird as the one responsible for the greatest proportion of the flock's movement decisions is likely to be beneficial as it will increase the flock's navigational accuracy. However, why leadership ranks in the rest of the flock do not segregate between experienced and inexperienced birds presents an interesting puzzle. Does the fact that the remaining experienced birds were not clustered in the top half of the hierarchy suggest a sub-optimal arrangement? Empirically, we are unable to compare the navigational efficiencies of flocks with and without such segregation, since we cannot impose the segregated (or ‘stratified’ (*sensu* [38])) structure on the group ourselves. However, previous modelling work provides some important insights into the potential benefits of embedding naive individuals among those better informed. First, agent-based simulations have suggested that merit-based stratification (which, in our case, we can interpret as sorting according to navigational experience) ultimately reduces the efficiency of information flow within networks of interacting individuals through the formation of ‘elites’ who preferentially associate with each other rather than with those outside their subgroup [39]. Second, adding uninformed individuals to groups can promote consensus decisions by ‘dampening’ conflict among subsets of individuals [40]. While such conflict in our flocks is likely to have been low since all our experienced flock members had undergone training together, it is nonetheless possible that a form of buffering by naive birds can enhance group performance within a hierarchical network. Future work into understanding not only the mechanisms through which certain individuals are placed in leadership positions but also how uninformed individuals affect information flow through networks they share with knowledgeable group mates will further inform us about the adaptive significance of hierarchical structures in decision-making by animal collectives.

## Data accessibility

Data available from the Dryad Digital Repository: http://dx.doi.org/10.5061/dryad.f9n8t.
